# Foveolar thickness as potential standardized structural outcome measurement in studies of Bietti crystalline dystrophy

**DOI:** 10.1038/s41598-022-16563-y

**Published:** 2022-08-29

**Authors:** Laura A. Jenny, Pei-Kang Liu, Masha Kolesnikova, Jimmy Duong, Angela H. Kim, Sarah R. Levi, Vivienne C. Greenstein, Stephen H. Tsang

**Affiliations:** 1 Edward S. Harkness Eye Institute, New York-Presbyterian Hospital, New York, NY USA; 2 Jonas Children’s Vision Care and Bernard & Shirlee Brown Glaucoma Laboratory, Department of Ophthalmology, Columbia University, New York, NY USA; 3 Department of Pathology and Cell Biology, Columbia University Irving Medical Center, New York, NY USA; 4 Institute of Human Nutrition, Columbia University, New York, NY USA; 5 Columbia University Stem Cell Initiative, New York, NY USA; 6 Department of Ophthalmology, Kaohsiung Medical University Hospital, Kaohsiung Medical University, Kaohsiung, Taiwan; 7 School of Medicine, College of Medicine, Kaohsiung Medical University, Kaohsiung, Taiwan

**Keywords:** Clinical genetics, Medical research, Outcomes research

## Abstract

Bietti crystalline dystrophy (BCD) is an ultra-rare orphan disorder that can lead to blindness. Because of the variable rates of progression of the disease, it is necessary to identify suitable outcome measurements for tracking progression in BCD. A retrospective analysis of patients with a clinical and genetic diagnosis of BCD was conducted. Four measurements of spectral domain-optical coherence tomography were compared to patients’ best corrected visual acuity. We observed that patients with higher measurements of foveolar thickness, choroidal thickness in the foveolar region, ellipsoid zone band length and the outer nuclear layer + area, had on average better visual acuity. Future studies are needed to validate the structural–functional correlations we observed in BCD and to propose a sensitive and clinically meaningful outcome measurement for tracking this rare, variable disease.

## Introduction

Bietti crystalline dystrophy (BCD; OMIM 210370) is an inherited retinal disorder that causes crystalline deposits in the retina and corneal limbus, retinal pigment epithelium (RPE) atrophy and choroidal sclerosis^1^. BCD typically results in symptoms of nyctalopia, and paracentral scotoma that both tend to begin manifesting in individuals in their twenties to forties. In the later decades, most patients with BCD develop peripheral visual field loss and visual impairment, which tend to cause legal blindness in patients aged 50 and older^2^. BCD is caused by mutations in the *CYP4V2* gene, which encodes a protein that is a member of the cytochrome P450 gene family^3–6^. It is an autosomal recessive dystrophy and is most common in patients of East Asian descent. However, BCD cases have also been documented in patients of European, North American, South American, Middle Eastern and African descent^5^. The disease is characterized by the formation of yellowish crystals in the retina, particularly in the posterior pole region of the eyes, and cornea^7^.

While the characterization of BCD is quite distinct, there is variability in disease progression^3,8^. Some of the key features of BCD progression are inter-eye symmetry, choroidal thinning, crystalline deposits, and ellipsoid zone (EZ) band disruption^1,9,10^. These features have been used to quantify progression; however, they have limitations. For example, measurement of the length of the EZ band may be useful in the early stages of the disease, but at later stages of the disease process, the EZ band may be so disrupted that it becomes difficult to measure; similarly calculation of the number of crystalline deposits may be useful in the early stages of BCD but increased RPE degeneration with disease progression results in loss of the crystals^3,11^. However, choroidal thinning may serve as a quantifiable measurement of progression, and in this study we included it as another potential structural outcome measurement. Currently, clinicians still lack easily measurable and reliable indicators that can be used to track the progression of BCD. In order to better treat and follow patients, a standard, easily interpretable quantitative measurement that correlates with best corrected visual acuity (BCVA) is needed. Thus, in this study, we analyzed four separate measurements (foveolar thickness, choroidal thickness, outer nuclear layer + (ONL +) area and EZ band length) on spectral domain-optical coherence tomography (SD-OCT) imaging and compared them to the patients’ BCVAs.

## Methods

### Subjects

Retrospective analysis was performed on 12 patients with a diagnosis of BCD and mutations in *CYP4V2*. All patients were evaluated at the Edward S. Harkness Eye Institute at Columbia University Irving Medical Center. Patient information was deidentified for use during analysis. No informed consent was needed given the retrospective study design, minimal risk conferred on the patients and the deidentification of all patient data. The study was performed under the Columbia University Institutional Review Board Approved Protocol AAAR8743. All procedures followed the tenets of the Declaration of Helsinki.

We analyzed results obtained from six patients with a clinical and genetic diagnosis of BCD. Of the original group of 12 patients in our database with BCD, six were excluded because one or more data values were missing in their electronic medical record. The number of visits for the six patients included in this study ranged from a minimum of one to a maximum of ten over a period of more than 5 years (Table 1). Visits in which cystoid macular edema (CME) was present in the foveal region were excluded from the analysis, as macular edema interferes with the accurate measurement of the retinal layers. The measurements of one eye from each of the two visits from patient 6 were excluded from analysis due to edema across the foveola (see Supplementary Fig. 1).

**Table 1:** Patient details (P1–P6).

Patient	Gender	Number of visits	Age at first visit	Genetic results
P1	M	10	29.8	c.802-8_810del17insGC, c.1062dupA
P2	F	2	35.7	c.219 T > A (p.F73L), c.992A > C (p.H331P)
P3	M	4	63.0	c.802-8_810del17insGC
P4	M	2	27.0	c.214G > C, CYP4V2 partial deletion of exon 7
P5	M	1	43.5	c.802-8_810del17insGC
P6	F	1	28.7	c. 254 G > A in exon 2, p. R85H

Four of the patients (P1, P2, P3, P4) had multiple visits and two patients (P5, P6) had a single visit examined in the study. The mean age of patients was 38 (sd = 12.5). Two of the patients were female and four of the patients were male.

### Clinical examination and imaging

All patients underwent a complete ophthalmic examination including fundoscopy and measurement of the BCVA at each visit. The measurement of BCVA was taken before dilation with tropicamide (1%), and phenylephrine (2.5%) as previously described^12,13^. All patients received spectral domain-optical coherence tomography (SD-OCT) imaging using Spectralis HRA + OCT (Heidelberg Engineering, Heidelberg, Germany). Horizontal SD-OCT scans (9 mm, 870 nm) were obtained with corresponding near infrared reflectance (NIR-R) (820 nm) fundus images. To obtain the highest signal-to-noise ratio for autofluorescence (AF) and OCT images, the eye-tracking function was used. Short-wave autofluorescence (SW-AF, 488 nm excitation, barrier filter transmitted light from 500 to 680 nm, 30° × 30°) was acquired using Spectralis HRA + OCT (Heidelberg Engineering, Heidelberg, Germany).

Wide-angle color fundus photos were obtained using an Optos 200 Tx unit (Optos; PLC, Dunfermline, United Kingdom).

### OCT procedure and segmentation

**Figure 1:**
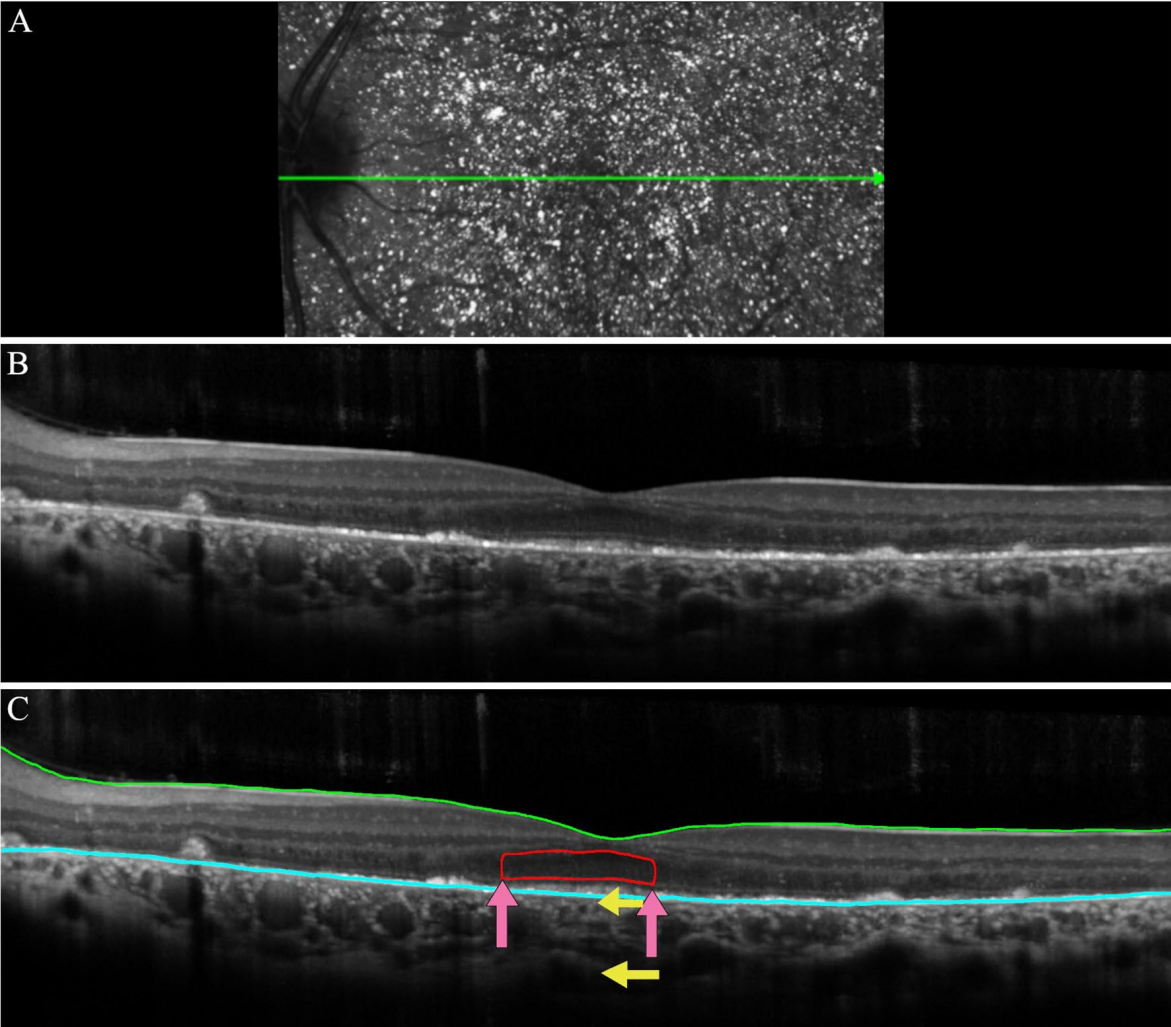
Spectral domain-optical coherence tomography (SD-OCT) imaging for patient 4 (P4). (**A**) The fundus view of the horizontal scan through the fovea of the spectral domain-optical coherence tomography (SD-OCT) for P4. The green line in the IR-R image indicates the position of the SD-OCT scan (**B**) Horizontal foveal scan. (**C**) A segmented line scan where the green line indicates the internal limiting membrane. The blue line indicates Bruch’s membrane. The red outlined portion is the outer nuclear layer + area (ONL + area). The pink arrows indicate the extent of the intact EZ band. The yellow arrows indicate the top and bottom of the choroid where the thickness was measured.

Horizontal SD-OCT scans (9 mm, 870 nm) with corresponding near NIR-R (820-nm) fundus images were analyzed. The automated segmentation of the internal limiting membrane and Bruch’s membrane were checked and manually corrected by two experienced observers, as previously described by Hood et al.^14,15^. After correction of the segmentation, the thicknesses at the foveola and fovea, as measured by Spectralis software, were recorded. For this study, the fovea was defined as 1 mm in diameter and the foveola was taken as the automated measurement of the center point on the thickness chart, as marked in Spectralis (Supplementary Fig. 2). Where possible, the length of the EZ band, the ONL + area, and the thickness of the choroid in the foveolar region, were measured as shown in Fig. 1. The ONL + area is defined as the area that extends from the outer plexiform layer border to the outer limiting membrane as defined by Hood et al*.*^15,16^. The averaged values for each of the measured variables obtained by the two observers were used in the statistical analysis.

### Statistical analysis

We performed statistical analysis on our dataset, which includes only 6 patients. Given the limitations of such a small dataset we present this information as supplemental to our main findings (see Supplementary Information).

## Results

### Patient demographics and clinical characteristics

The clinical characteristics of the 6 patients aged 27 to 63 are shown in Table 1. All patients had complaints of decreased visual acuity.

**Figure 2:**
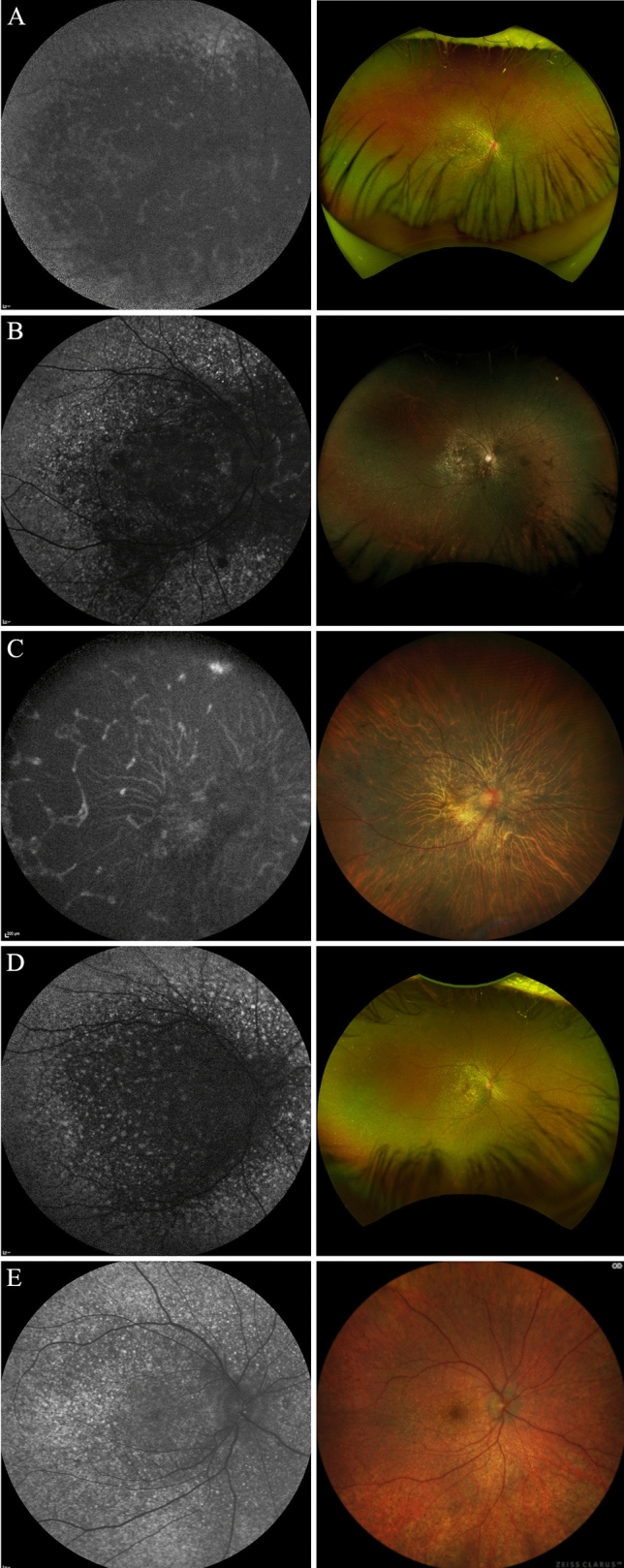
Short-wave autofluorescence (SW-AF) and color fundus photos of five patients with BCD. (**A–E**) Left column: SW-AF images reveal RPE atrophy. (**A,B,D,E**) Right column: color fundus images demonstrate yellow-white, shiny crystalline deposits in the posterior pole of patients 1, 2, 4, and 6, respectively.

The average age of the patients at the first examination was 38 (± 12.59; range 27 to 63) years. Mean foveolar thickness for normal controls is reported to be 182 ± 23 μm^17^. The foveolar thickness for the patients analyzed in this study ranged from 96 μm to 250 μm. Color fundus photography for P1, P2, P4, and P6 revealed crystalline deposits in the posterior pole of the retina (Fig. 2A,B,D,E). P3 and P5 did not have crystalline deposits present, characteristic of later stage BCD^18^. SW-AF revealed RPE atrophy (Fig. 2A–E) for all patients examined. P6 presented with severe edema in the foveola and foveal region of the left eye at visit 1. P5 did not have SW-AF or color fundus photography available for presentation but had SD-OCT and BCVA measurements which were analyzed.

### Structural measurements and visual acuity

**Figure 3:**
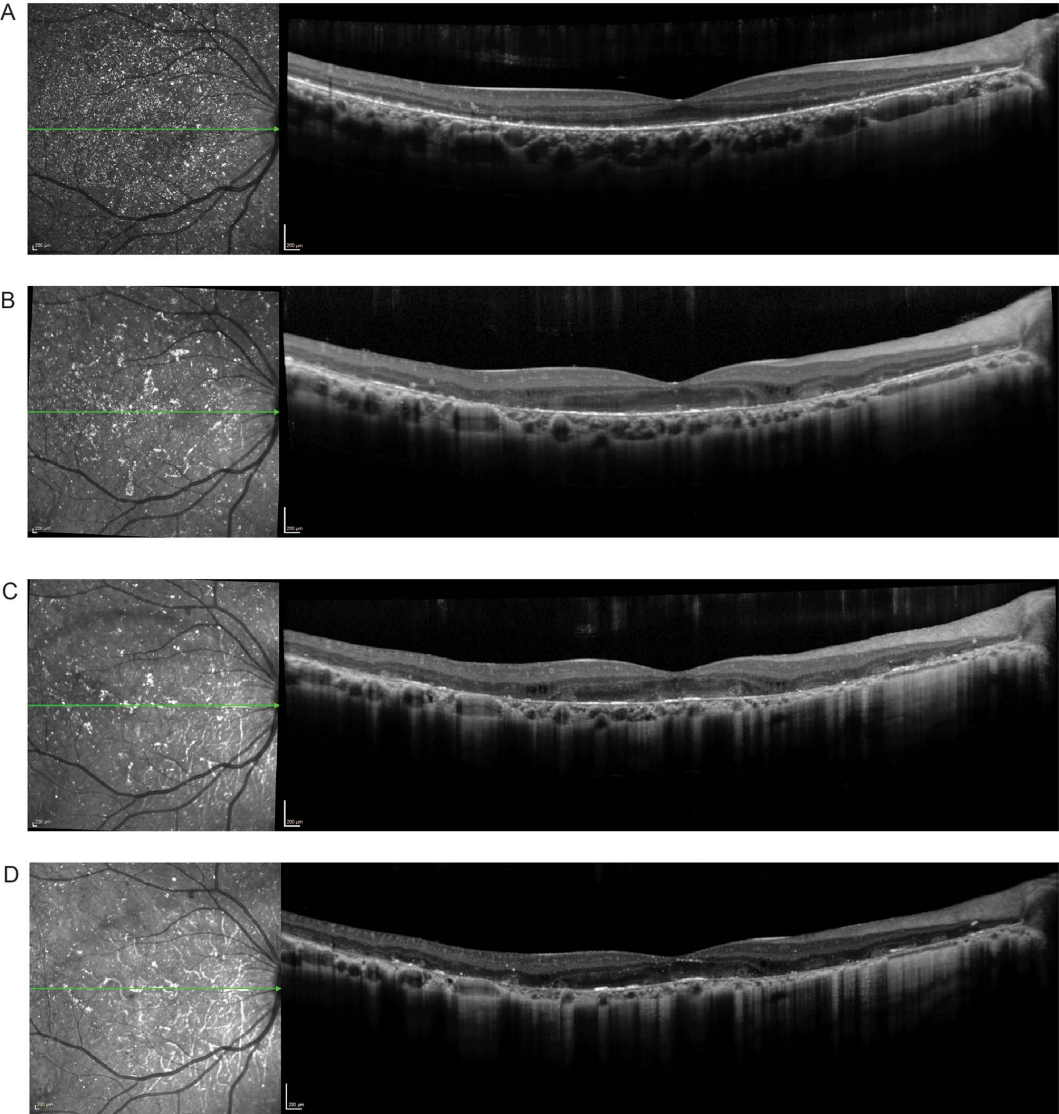
Spectral domain-optical coherence tomography (SD-OCT) for P1. The green lines in the IR-R images indicate the position of the SD-OCT scans. (**A**) Horizontal scan through the fovea of P1 shows a preserved EZ band at the fovea at initial presentation. The horizontal scan through the fovea 3 (**B**), 4 (**C**), and 5 (**D**) years after initial presentation demonstrates disruption and loss of the EZ band, and progressive shrinking of the ONL + area, along with decreased foveolar and choroidal thickness.

P1 presented with a BCVA of 20/30 (logMAR = 0.176) in the right eye which decreased to 20/60 (logMAR = 0.477) over a 6-year period. There was progressive degeneration of the EZ band, coupled with foveola thinning and shrinking of the ONL + area. Choroidal thickness also decreased over the 6-year period (Fig. 3).

**Figure 4:**
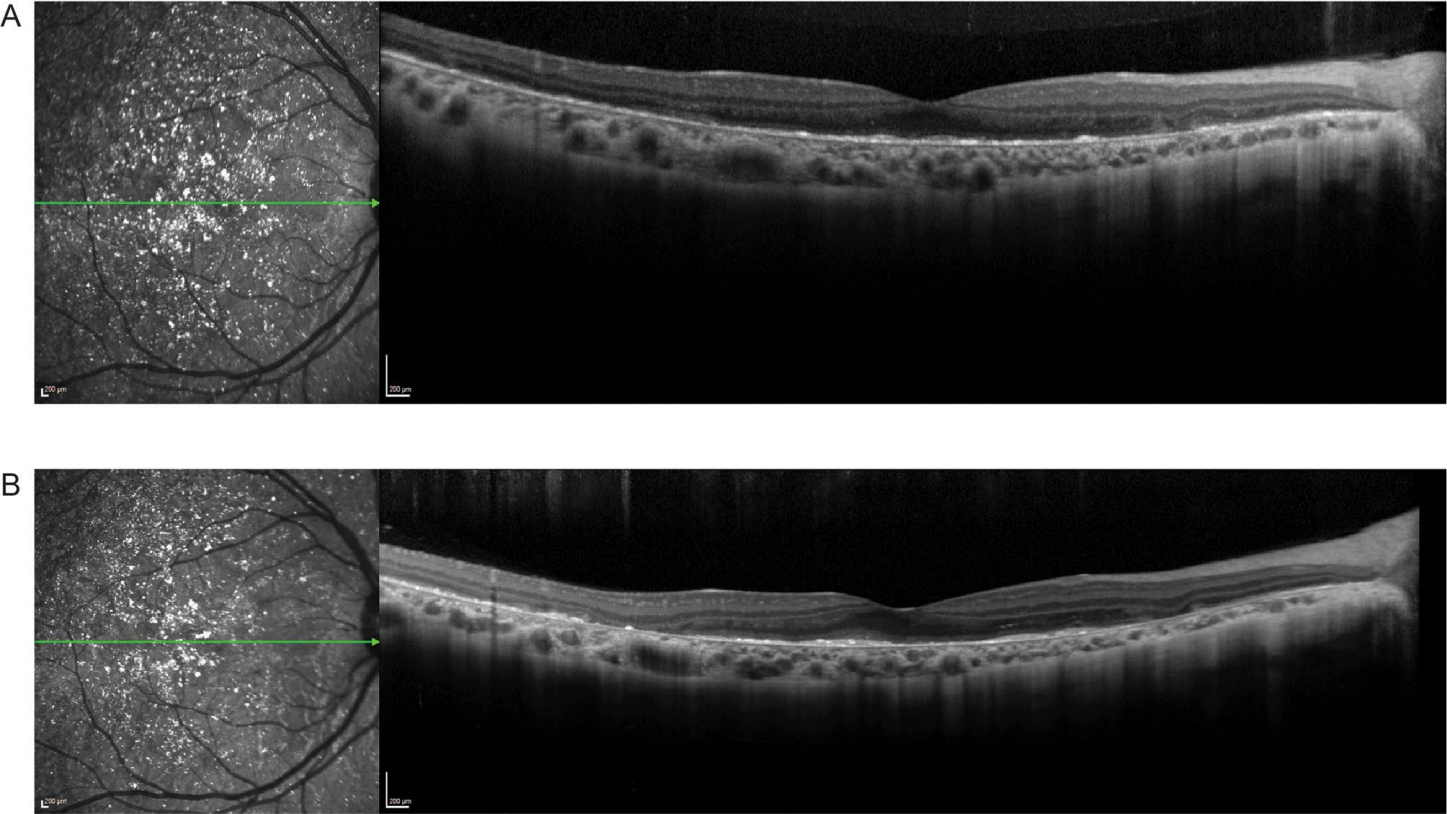
Spectral domain-optical coherence tomography (SD-OCT) for P2. The green lines in both IR-R images indicate the position of the SD-OCT scans. (**A**) The horizontal scan through the fovea of the right eye for P2 at initial presentation and (**B**) the one year follow up shows progressive loss of the EZ band and retinal layer structure, as well as slightly decreased foveolar thickness, ONL + area, and choroidal area.

P2 also demonstrated loss of visual acuity and progressive degeneration of the EZ band. The patient presented with an initial BCVA of 20/32 (logMAR = 0.2) in the right eye which decreased to 20/60 (logMAR = 0.477) after a year. The decrease in BCVA was accompanied by loss of the EZ band, but only minor changes in the ONL + area, foveola thickness and choroidal thickness. This case provides an example of the relevance of measurements of EZ band length in predicting visual acuity in patients with BCD (Fig. 4).

**Figure 5:**
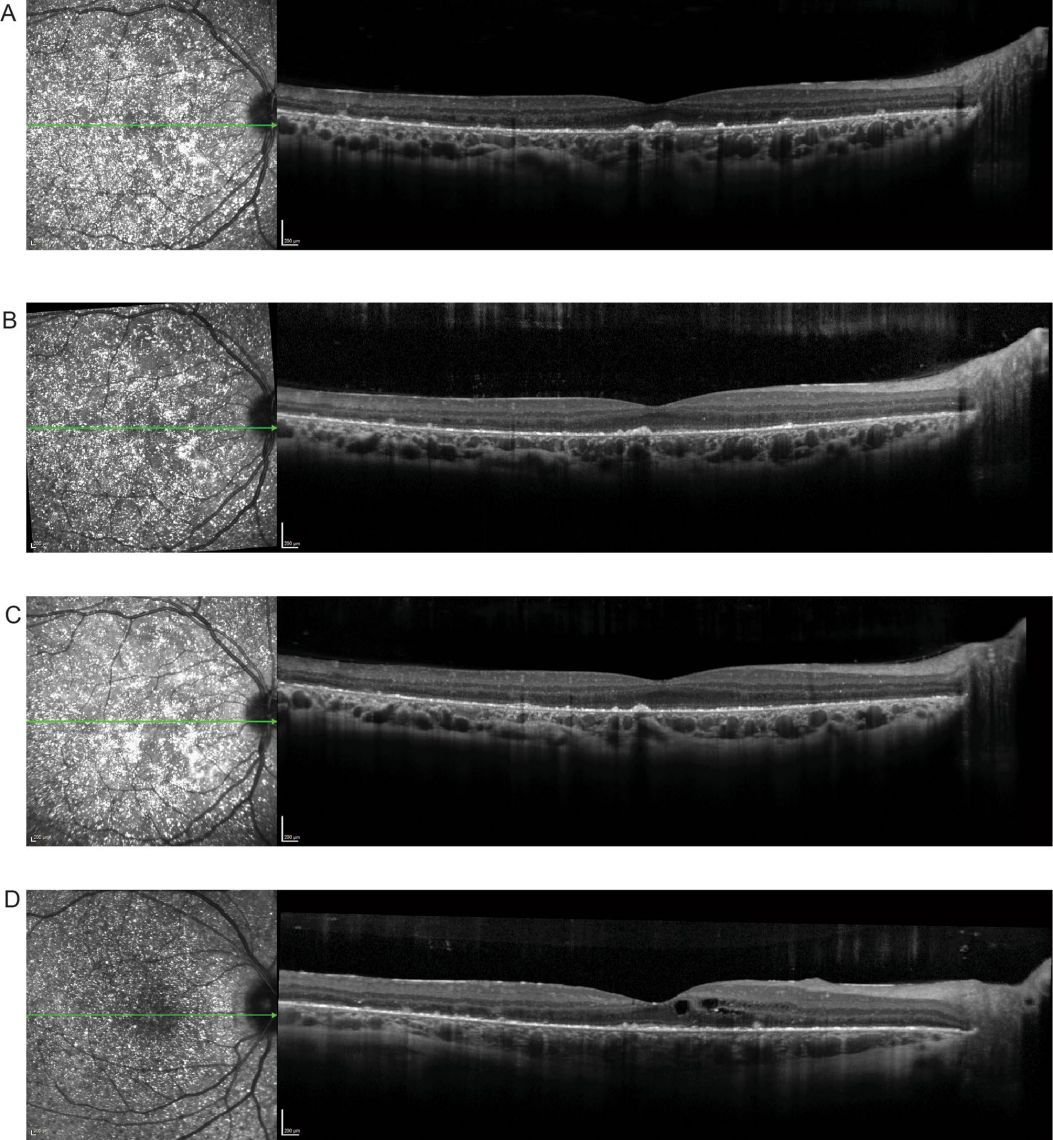
Spectral domain-optical coherence tomography (SD-OCT) for P4 and P6. **(A)** The horizontal scan through the fovea of the right eye for P4 at initial presentation in our clinic, **(B)** at a 2-month follow up visit and **(C)** at a 4-month follow up visit showed maintenance of the retinal layer structure and minor changes in the ONL + area, choroidal thickness, and foveolar thickness. **(D)** The horizontal scan through the fovea of the right eye of P6 shows an intact EZ band and the beginning of parafoveal cystoid macular edema. The green line in each IR-R image indicates the position of the SD-OCT scan.

P4 and P6 are examples of patients at early stages of BCD with good visual acuity. P4 had 20/30 (logMAR = 0.176) vision in the right eye and a preserved EZ band. P6 had 20/25 (logMAR = 0.097) vision in the right eye and similarly had a preserved EZ band. Visual acuity, EZ band length, ONL + area, foveola thickness and choroidal thickness for P4 did not change for the three visits which spanned a period of 8 months (Fig. 5). We did not include progression of P6 as severe edema developed in the right eye after the first visit (Supplemental Fig. 1).

**Figure 6:**
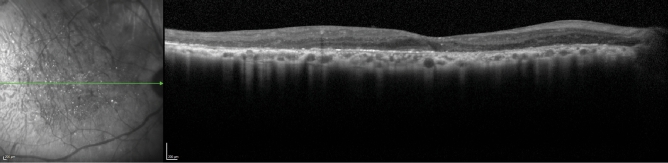
Spectral domain-optical coherence tomography (SD-OCT) for P5 showing a preserved EZ band. The green line in the IR-R image indicates the position of the SD-OCT scan.

P5 presented with a visual acuity of 20/50 (logMAR = 0.398) in the right eye at the first visit along with a preserved EZ band. The patient did not return for follow up visits (Fig. 6).

**Figure 7:**
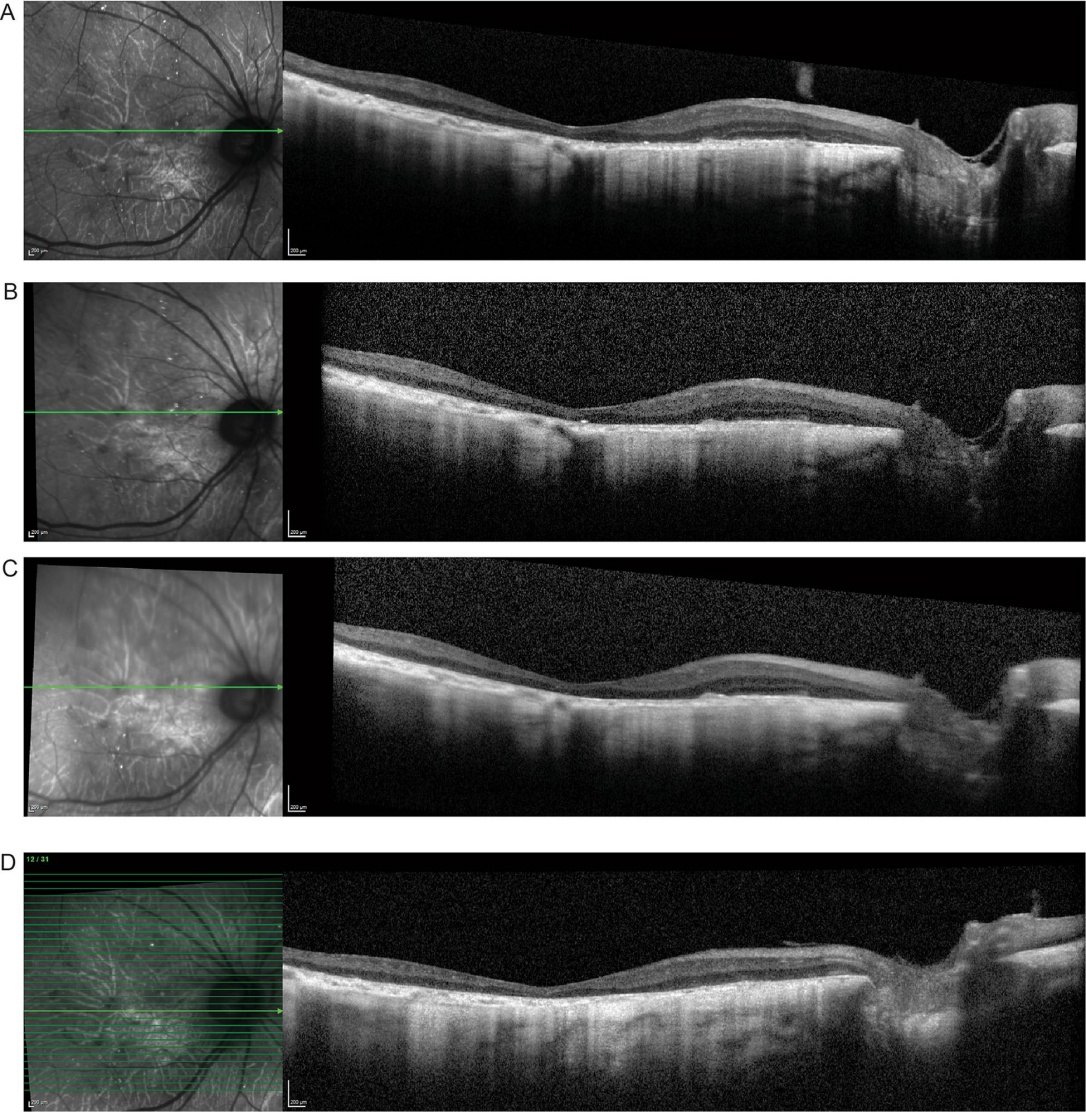
Spectral domain-optical coherence tomography (SD-OCT) for P3. The green line in each IR-R image indicates the position of the SD-OCT scan. (**A**) The horizontal scan through the fovea of the right eye for P3 at initial presentation (**B**) at 3-month follow up visit (**C**) at 6-months and (**D**) a 3-year follow up visit showing absence of EZ band, loss of the retinal layer structures and progressive decrease of foveolar thickness.

However, for patients at advanced stages of the disease it may not be possible to measure the EZ band, as it may be absent. An example is P3 who presented with a visual acuity of count fingers at three feet and with complete absence of the EZ band in the right eye (Fig. 7). Over the course of two years visual acuity decreased to hand motion and foveolar and choroidal thickness decreased.

## Discussion

We investigated possible outcome measures in a small group of patients with BCD. This preliminary investigation sought to assess structural measurements and relate them to visual acuity. One challenge in treating rare inherited genetic disorders is identifying suitable structural outcome measurements that can be used to track disease progression. As clinicians move forward with clinical research trials of BCD^19,20^, identifying a reliable, quantifiable, and clinically meaningful outcome measurement is key for clinical trials to commence. While the FDA recommends a 15-letter change in visual acuity as a clinically relevant outcome measurement, other more sensitive measures of change in patients are being explored for use in various clinical trials^21^. The coupling of structural measures to functional measures may serve as more sensitive indicators to monitor the stages of disease in patients^9^.Several studies have proposed the use of structural outcome measurements in correlation with visual acuity^22–24^. While structural measurements may be more sensitive to changes in the retina during disease progression, investigations on the structure–function relationships must be conducted to be able to ensure structural measures are clinically meaningful.

The four structural measurements we investigated (foveolar thickness, choroidal thickness in the foveolar region, ONL + area and EZ band length) were related to visual acuity (see supplementary Fig. 3, Fig. 4, Supplementary Information Tables 1, 3 and 4). We suggest that of these four measurements, foveolar thickness may be considered as a potential structural outcome measurement for patients with BCD for the following reasons. For late-stage patients, foveolar thickness may be one of few remaining measurable structural characteristics. It can be automatically measured using Spectralis software and standardized across different clinical sites. Moreover, when applied to multi-center clinical trials/studies, OCT scans allow for maximal standardization, thereby minimizing bias between different sites. Identifying suitable functional and structural measurements that can be used to track disease progression is of high importance for future clinical trials^9,25^. The work of Hood et al. has emphasized the importance in standardizing and verifying the accuracy of automated segmentation of OCT scans^14,15,26^.

The other structural measurements we investigated (ONL + area, choroidal thickness in the foveolar region, and EZ band length) have the following limitations. For most patients with mid- to late-stage BCD, identifying the border of the ONL + layer is difficult, as degeneration of the retina leads to poor visualization of the outer plexiform layer and the outer limiting membrane. A drawback of choroidal thickness is that it is not automatically measured in the Spectralis software, so researchers need to pay close attention to their methods of segmenting and measuring choroidal thickness. Typically, two graders are used to measure the choroidal thickness. While this method is widely used, it may be prone to human error, resulting in problems with measurement reliability. Lastly, the measurement of EZ band length is a promising strategy to explain structure and function correlation. EZ band length is an established predictor of visual function, and for patients at the early stages of the disease this measurement may provide more insight into how retinal structure correlates with the functional vision of the patient^27^. However, disruption of the EZ band is common in BCD patients and for most late-stage patients, the EZ band is absent. For these reasons, use of EZ band length as a structural outcome measure may be challenging.

Future clinical trials may seek to use a combination of structural measurements to track BCD progression with greater sensitivity. Measurements of foveolar thickness at only a single point in time may not provide clinical trialists with a useful indicator of visual function, especially for patients at advanced stages of disease when visual acuity has declined to hand motion or light perception. Rather, measurements of the decreases in foveolar thickness over time may be more useful to track the progression of BCD. When used in combination with EZ band length, which is an established structural measurement used to predict function, it may help provide clinicians with a more sensitive measurement of structural and functional changes. Foveolar thickness may be particularly helpful to provide more information for patients in mid-stages of the disease where EZ band disruption makes it hard to measure the EZ band length.

Aside from these structural measurements, microperimetry has been proposed as a potential method for assessing visual function in BCD patients^8^, but for late-stage patients, microperimetry testing may be of limited value if visual acuity is very poor and fixation is unsteady. For trials of BCD patients at late stages of disease, mobility testing may be considered as an outcome measurement^28^.

BCD is an ultra-rare inherited genetic disorder^29^, limiting our ability to analyze a large number of patients. The main limitations of this study are the small sample size and the random follow-up time^30^.

Further studies need to be done to confirm our observations in a larger cohort of BCD patients who have been followed over several time points. Here, we have demonstrated how SD-OCT measurements of retinal structure may be applied to future datasets of patients with BCD to help provide the structure–function relationships which may assist in the selection of a reliable and clinically accurate outcome measurement in BCD.

## Supplementary Information

Supplementary Figure 1.

Supplementary Figure 2.

Supplementary Figure 3.

Supplementary Figure 4.

Supplementary Information.

## Data Availability

All de-identified data will be publicly made available upon reasonable request from the authors. Data analysis results and SPSS scripts are attached in supplementary file 1.
