# Supplementary figures and images for: Foveolar thickness as potential standardized structural outcome measurement in studies of Bietti crystalline dystrophy
Source: Sci Rep. 2022 Aug 29;12:14706. doi: 10.1038/s41598-022-16563-y (PMC9424222; doi:10.1038/s41598-022-16563-y)

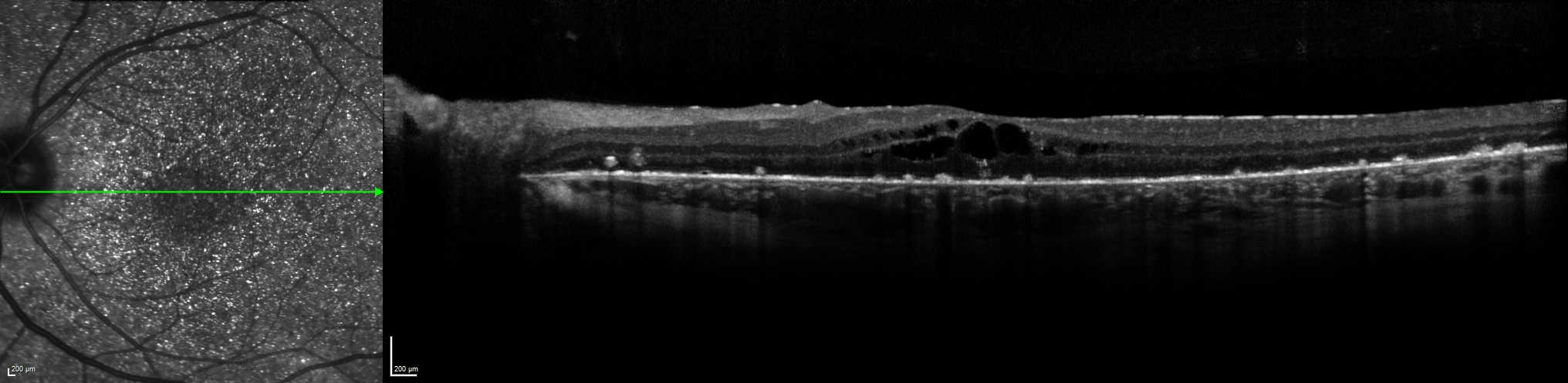

Supplement: Supplementary file 1 — Supplementary Figure 1. [file 41598_2022_16563_MOESM1_ESM.jpg]

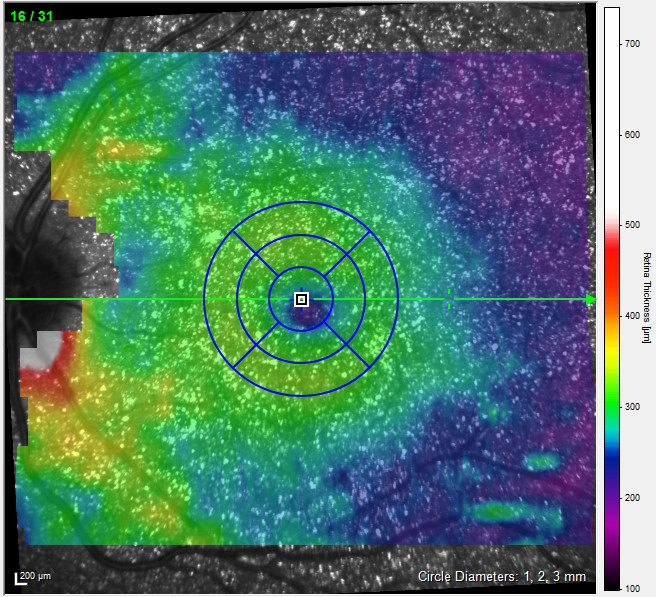

Supplement: Supplementary file 2 — Supplementary Figure 2. [file 41598_2022_16563_MOESM2_ESM.png]

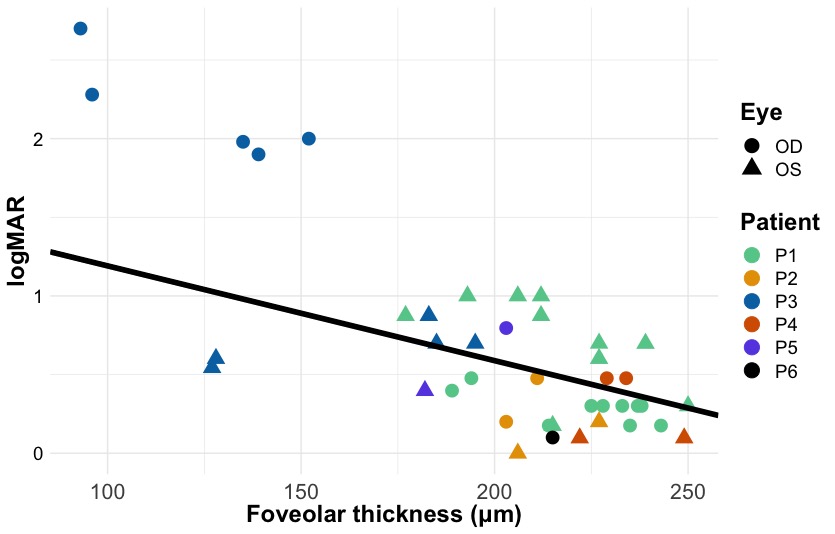

Supplement: Supplementary file 3 — Supplementary Figure 3. [file 41598_2022_16563_MOESM3_ESM.jpeg]

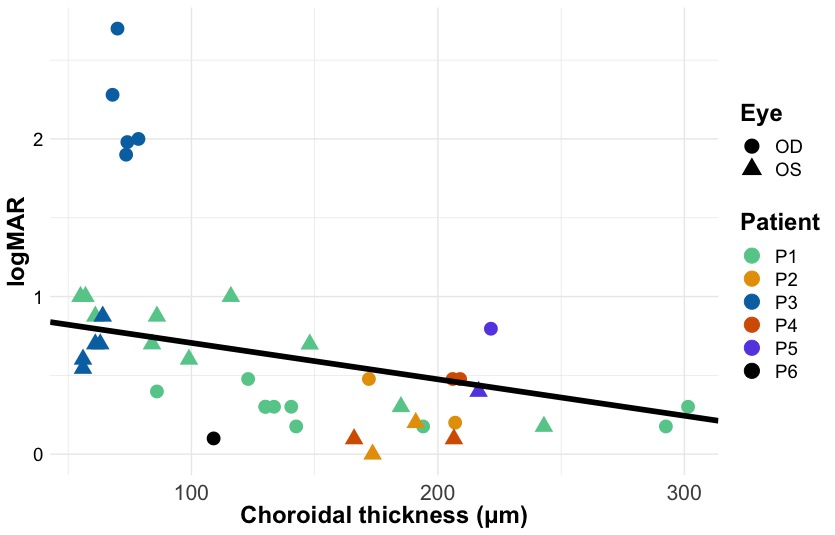

Supplement: Supplementary file 4 — Supplementary Figure 4. [file 41598_2022_16563_MOESM4_ESM.jpeg]
