# Supplementary material for: Foveolar thickness as potential standardized structural outcome measurement in studies of Bietti crystalline dystrophy
Source: Sci Rep. 2022 Aug 29;12:14706. doi: 10.1038/s41598-022-16563-y (PMC9424222; doi:10.1038/s41598-022-16563-y)
Supplement: Supplementary file 5 — Supplementary Information. [file 41598_2022_16563_MOESM5_ESM.docx]

Note on Statistical Analysis:

We performed statistical analysis on our dataset to examine the relationship between visual acuity and structural measurements, which includes only 6 patients. Given the limitations of such a small dataset we present this information as supplemental to our main findings.

Statistical Analysis Method

BCVA was converted to a logarithm of the minimum angle of resolution (logMAR). For patients who had a BCVA of count fingers at one foot, a logMAR value of 1.98 was used as described in previous studies^14-16^. For patients who had a BCVA of count fingers at three feet, a logMAR value of 1.9 was assigned^17^. Lastly, for patients who had a BCVA of hand motion, a logMAR value of 2.7 was assigned^15,16^.

Statistical analysis was performed in SPSS Statistics Version 28.0.0. The dataset had repeated measurements, because data from both eyes was included and some patients had measurements from multiple visits. To account for correlation among observations in the data, we fit linear mixed models^21-23^. The outcome in these mixed models was BCVA in logMAR. Separate mixed models were fit to examine the relationship between logMAR and the following structural variables: the foveolar thickness (μm), the ONL+ area (μm^2^), length of the EZ band (μm), and choroidal thickness (μm).

Results of Statistical Analysis

Linear mixed models were fit to examine the relationship between logMAR and the structural measurements. For all structural measurements, we observed a negative relationship, whereby lower values of the structural measurements (decreased thickness, length or area), corresponded with higher values of logMAR, i.e. poor/decreased visual acuity. We observed that average logMAR values tend to be lower for higher values of foveolar thickness (slope = -0.006, se = 0.002, p-value = <0.001) (Supplementary Figure 3, Table 2). Similarly, average logMAR values tend to be lower for higher values of choroidal thickness in the foveolar region (slope=-0.002, se=0.0007, p-value=0.002) (Supplementary Figure 4 Table 3), EZ band length (slope=-0.0001, se=0.00005, p-value=0.009) (Table 4), and the ONL+ area (slope = -1.7, se=0.6, p=0.012) (Table 5). We did not fit a mixed model to examine the relationship between logMAR and foveal thickness, since foveal thickness can be influenced by edema in any part of the fovea.

| Parameter | Estimate | Std. Error | Sig. |
| --- | --- | --- | --- |
| Intercept | 1.795 | 0.356 | <0.001 |
| Foveolar Thickness (μm) | -0.00603 | 0.00162 | <0.001 |

Table 1: Linear mixed model where the outcome was best corrected visual acuity (BCVA, **logMAR)** and the predictor was foveola thickness (μm). Decreased foveolar thickness had on average increased logMAR which signifies poor visual acuity .

| Parameter | Estimate | Std. Error | Sig. |
| --- | --- | --- | --- |
| Intercept | 0.937 | 0.204 | <0.001 |
| Choroidal Thickness (μm) | -0.00231 | 0.000695 | 0.002 |

Table 2: Linear mixed model where the outcome was best corrected visual acuity (BCVA, **logMAR)**  and the predictor was the choroidal thickness under the foveola (μm). Decreased subfoveolar choroidal thickness had on average increased logMAR which signifies poor visual acuity .

| Parameter | Estimate | Std. Error | Sig. |
| --- | --- | --- | --- |
| Intercept | 0.794 | 0.190 | <0.001 |
| EZ length (μm) | -0.00131 | 0.000047 | 0.009 |

Table 3: Linear mixed model where the outcome was best corrected visual acuity (BCVA, **logMAR)**  and the predictor was ellipsoid zone (EZ) band length (μm). Decreased EZ length had on average increased logMAR which signifies lower worse visual acuity .

| Parameter | Estimate | Std. Error | Sig. |
| --- | --- | --- | --- |
| Intercept | 0.975 | 0.216 | <0.001 |
| ONL+ area (μm^2^) | -1.712 | 0.647 | 0.012 |

Table 4: Linear mixed model where the outcome was best corrected visual acuity (BCVA, **logMAR)** and the predictor was the outer nuclear layer+ (ONL+) area (μm^2^). Decreased ONL+ area had on average increased logMAR which signifies poor visual acuity.
